# A Comparative Evaluation of Different Irrigation Activation Techniques on Root Canal Cleanliness: A Scanning Electron Microscope Study

**DOI:** 10.1055/s-0044-1801306

**Published:** 2025-03-06

**Authors:** Lubna Ahmad, Prashant Bhasin, Hemanshi Kumar, Vrinda Vats, Prateek Madan, Monika Tandan

**Affiliations:** 1 Department of Conservative Dentistry and Endodontics, Sudha Rustagi College, Pandit Bhagwat Dayal Sharma University of Health Sciences, Faridabad, Haryana, India; 2 Department of Conservative Dentistry and Endodontics, Manav Rachna Dental College, Manav Rachna International Institute of Research and Studies, Faridabad, Haryana, India

**Keywords:** diode laser, root canal cleanliness, scanning electron microscope, sonic, ultrasonic activation

## Abstract

**Objective:**

This article compares the effect of ultrasonic, sonic activation of intracanal heated 3% sodium hypochlorite and laser-activated 3% sodium hypochlorite (NaOCl) on root canal cleanliness using scanning electron microscope.

**Materials and Methods:**

Eighty-eight permanent mandibular premolars were extracted and decoronated to obtain 14 mm of standardized root length. Working length was calculated and canals were prepared till size 25/0.04. Samples were randomly divided into four groups according to the method of irrigation employed ( *n* = 22)—group A: passive ultrasonic irrigation (PUI) of 3% intracanal heated NaOCl, group B: sonic activation of 3% intracanal heated NaOCl, group C: activation of 3% NaOCl by diode laser, and group D: conventional needle irrigation (CNI). Samples were split into two halves and the presence of debris at these regions was graded under scanning electron microscope. The chi-square test was employed to assess significant differences in cleanliness scores and pairwise comparisons using the Dunn test were performed to identify specific group difference. A *p* -value of < 0.05 was kept as level of significance for all analysis.

**Results:**

Group A and group B showed maximum cleanliness in middle third as compared with apical third. Group C (laser) showed better cleanliness in apical third as compared with middle third. While group D (CNI) irrigation showed the lowest cleaning efficiency both in the middle third and apical third.

**Conclusion:**

It was concluded that PUI with intracanal heating of 3% NaOCl and diode laser activation of 3% NaOCl can be recommended as a potential irrigant activation strategy to effectively clean inaccessible areas of the root canal system.

## Introduction

The success of endodontic treatment relies on the effective debridement of root canals and involves complete disinfection and removal of pulp tissue remnants and bacterial toxins from deep crevices. 1 Irrespective of the instrumentation used, certain areas go untouched and cause reinfection. Therefore, to achieve complete disinfection, chemomechanical preparation of the root canal system is done. Sodium hypochlorite (NaOCl) is considered as a gold-standard irrigant in endodontics. It is known for its pulp tissue dissolution ability in addition to its antimicrobial properties. 2

Among the different irrigation techniques, conventional needle irrigation (CNI) has been in use since decades but this technique generates inadequate shear stress and has low dentinal tubule penetration. 3 Ultrasonic activation remains the first and most widely used activation technique. It works at a frequency of 40 to 45 kHz. 4 Passive ultrasonic activation (PUI) is a noncutting technology that works by the phenomenon of rapid fluid movement in circular motions around the activation tip. 5 6

Another irrigation activation technique is the sonic activation. Sonic irrigation devices function at much reduced frequencies of 1,000 to 6,000 Hz, utilizing a flexible, noncutting soft polymer tip that is attached to a sonic handpiece. 7

High-power diode laser was pioneered in the mid-1900s in the field of dentistry. They are classically indicated in endodontics for photothermal disinfection. The wavelength mostly employed is 800 to 1,064 nm, which uses an optical fiber as a delivery system. 8

Increasing the temperature of NaOCl solution enhances its cleaning potential and has a decontamination effect on the root canal system. 9 NaOCl can be heated both extraorally as well as within the canal. Literature states that intracanal heating of NaOCl improves its immediate tissue-dissolution capacity and eliminates dentinal debris to a much larger extent. 10

Scanning electron microscope (SEM) is one such technology that allows visualization of images at a magnification of 50× to 10,000× and beyond. In endodontics, SEM is used mainly for topographic analysis and surface evaluation of dentin after different rotary instruments and techniques. 11

## Materials and Methods

Eighty-eight extracted permanent mandibular first premolars were selected with straight roots and single canal and fully formed apices. Teeth with root caries, calcified canals, immature open apices, root resorption, fractured teeth, or teeth subjected to previous endodontic treatment were excluded. The selected teeth were kept in normal saline until further use and decoronated by slitting the crown at the cementoenamel junction to a length of 14 mm.

## Endodontic Treatment

The canal patency was evaluated and working length was determined using #10-K file. Wax was used to seal the apical foramen of each root to simulate the *in vivo* apical counterpressure and avoid leakage of the irrigant through the apical foramen. Canals were instrumented with JIZAI rotary files with continuous motion at a rotation speed of 500 revolutions per minute in a sequence: 25/0.14, 13/0.04 till size 25/0.04. During instrumentation, canal was irrigated with 5 mL of 3% NaOCl, via a 30G side-vented needle in a disposable syringe, followed by rinsing with sterile saline; further irrigation with 3 mL of 17% ethylenediaminetetraacetic acid for 1 minute was performed to eliminate smear layer and at the end rinsed with 3 mL of sterile saline. An equal volume of 3% NaOCl was then injected into the root canals, using a 30G needle at 2 mm from the working length in all teeth.

The samples were randomly divided into four groups, according to the method of irrigation employed:

Group A ( *n* = 22): PUI of 3% intracanal heated NaOCl: Intracanal heating of 3% NaOCl was performed for 4 seconds with a heated plugger at 3 mm from the working length, at 180°C followed by ultrasonic activation. Irrigant was activated via an ultrasonic tip of size (25/0.02), 3mm from apex, mounted on ultrasonic activator. The solution was activated by agitating in short vertical strokes for 30 seconds. The activation sequence was 30 seconds of PUI, repeated three times with irrigant flooding.

Group B ( *n* = 22): Sonic activation of 3% intracanal heated NaOCl: Intracanal heating of 3% NaOCl was performed for 4 seconds, with a heated plugger at 3mm from working length, at 180°C followed by sonic activation by activator. The sonic tip (size 25/0.02) was moved in short 2 to 3 mm vertical strokes for 30 seconds. NaOCl was refreshed after this and the cycle was repeated thrice.

Group C ( *n* = 22): Activation of 3% NaOCl by laser: Specimens were treated with diode laser—980 nm (IMDSL) in continuous-wave mode, at a power setting of 0.6 W with a 200-µm fiber tip, which was introduced 1 mm short of the apex for 5 seconds and then repeated four times at an interval of 5 seconds. Specimens treated with diode laser do not require intracanal heating as laser generates heat itself.

Group D: CNI (control): 3% NaOCl was delivered through a disposable syringe and 30G side-vented needle.

Specimen preparation for SEM examination: Grooves were made along the surface of the specimens vertically and the specimens were then split longitudinally. The sections were dried at room temperature for 24 hours and then sputter-coated with 0.3-mm thick layer of chromium (Cr) in a sputter coater and sent for SEM analysis. 10

SEM examination: SEM images were taken of the samples at a standard magnification of ×1,000 at the middle and apical third of the root canal (from apex to 5 mm from head of sample). The presence of debris at these regions was graded using the rating system, proposed by Hülsmann et al 12 :

Score 1: Clean root canal walls with only a few small debris particles
Score 2: Few small agglomerations of debris
Score 3: Many agglomerations of debris covering < 50% of the root canal walls
Score 4: > 50% of the root canal walls covered by debris
Score 5: Complete or nearly complete root canal walls covered by debris

## Statistical Analysis

Sample size was estimated using GPower software (version 3.0). Sample size was estimated for *F* -tests—analysis of variance: fixed effects, omnibus, one way, for four groups, was chosen.

The chi-squared test was employed to assess significant differences in cleanliness scores among groups for both the middle and apical thirds. Descriptive statistics, including mean, standard deviation, median, and interquartile range, were computed to provide an overview of cleanliness score distributions within each group. Pairwise comparisons using the Dunn test were performed to identify specific group differences in cleanliness scores. Additionally, Pearson's correlation coefficients were calculated to explore associations between middle and apical cleanliness scores within each group. A *p* -value of < 0.05 was considered as level of significance for all analyses.

## Results

In the middle third, ultrasonic activation demonstrated notable success, with 63.6% of samples achieving the highest cleanliness score, indicating clean root canal walls ( Table 1 ). In contrast, group B had no samples with the highest cleanliness score, and 68.2% of samples showed many agglomerations of debris. Group C showed a moderate success rate, with 18.2% achieving the highest cleanliness score. Group D had no samples with the highest cleanliness score.

**Table 1:** Debris score comparisons between all groups in the middle and apical third

Middle third debris comparison							Apical third debris comparison					
Group	1	2	3	4	5	Total	1	2	3	4	5	Total
A	14 (63.6%)	8 (36.4%)	0 (0.0%)	0 (0.0%)	0 (0.0%)	22 (100.0%)	7 (31.8%)	12 (54.5%)	3 (13.6%)	0 (0.0%)	0 (0.0%)	22 (100.0%)
B	0 (0.0%)	7 (31.8%)	15 (68.2%)	0 (0.0%)	0 (0.0%)	22 (100.0%)	0 (0.0%)	2 (9.1%)	17 (77.3%)	3 (13.6%)	0 (0.0%)	22 (100.0%)
C	4 (18.2%)	17 (77.3%)	1 (4.5%)	0 (0.0%)	0 (0.0%)	22 (100.0%)	5 (22.7%)	10 (45.5%)	7 (31.8%)	0 (0.0%)	0 (0.0%)	22 (100.0%)
D	0 (0.0%)	0 (0.0%)	4 (18.2%)	10 (45.5%)	8 (36.4%)	22 (100.0%)	0 (0.0%)	0 (0.0%)	0 (0.0%)	2 (9.1%)	20 (90.9%)	22 (100.0%)
Total	18 (20.5%)	32 (36.4%)	20 (22.7%)	10 (11.4%)	8 (9.1%)	88 (100.0%)	12 (13.6%)	24 (27.3%)	27 (30.7%)	5 (5.7%)	20 (22.7%)	88 (100.0%)
Chi-square	129.7611						119.8074					
df	12						12					
*p* -Value	< 0.0001						< 0.0001					

Abbreviation: df, degrees of freedom.

In the apical third, group A showed 31.8%, achieving the highest cleanliness score, while 54.5% had few small agglomerations of debris (score 2). In group B, no samples achieved the highest cleanliness score, with 77.3% having many agglomerations of debris (score 3). Group C (laser activation) demonstrated 22.7%, achieving the highest cleanliness score and 45.5% with few small agglomerations of debris. Group D (CNI) had 90.9% of samples with score 5, indicating significant debris agglomerations.

## Intergroup Comparison

**Fig. 1:**
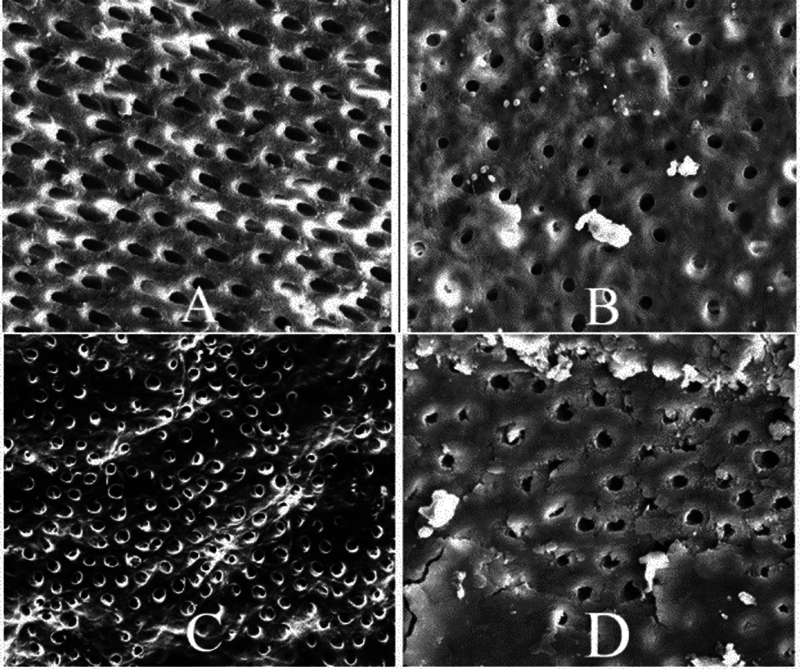
Root canal cleanliness in the middle third ( **A,** passive ultrasonic irrigation [PUI]; **B,** sonic; **C** , laser; and **D** , conventional needle irrigation [CNI]).

The results in the middle third showed that the maximum cleanliness was achieved by group A (PUI) > group C (laser activation) > group B (sonic activation) and the least cleanliness was achieved by group D (CNI) ( Table 2 , Fig. 1 ).

**Table 2:** Pairwise comparison between all groups in middle and apical third

Pairwise comparison of middle third score between all groups				Pairwise comparison of apical third score between all groups			
Groups	Average difference	Dunn test ( *Z* )	*p* -Value	Groups	Average difference	Dunn test ( *Z* )	*p* -Value
A - B	–2	–4.529292	< 0.001	A - B	–1	–3.7044329	0.001
A - C	–1	–1.689303	0.547	A - C	0	–0.8415008	1
B - C	1	2.839988	0.027	B - C	1	2.8629321	0.025
A - D	–3	–7.73652	< 0.001	A - D	–3	–7.3936213	< 0.001
B - D	–1	–3.207228	0.008	B - D	–2	–3.6891884	0.001
C - D	–2	–6.047217	< 0.001	C - D	–3	–6.5521205	< 0.001

When comparing group A (ultrasonic activation) and group B (sonic activation), PUI showed significant cleanliness with middle third. Similarly, when contrasting group A with group C, PUI resulted in more effective cleanliness compared with laser activation with a nonsignificant *p* -value of 0.547.

While comparing group B and group C, laser activation was more efficient in achieving cleaner middle thirds compared with sonic activation with a significant *p* -value of 0.02.

**Fig. 2:**
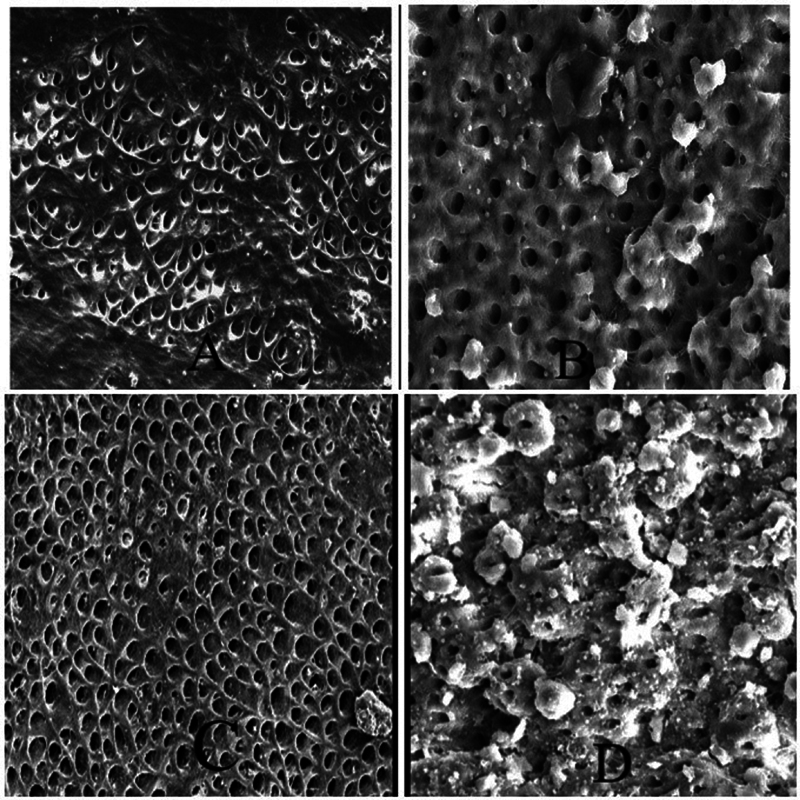
Root canal cleanliness in the apical third ( **A** ), passive ultrasonic irrigation (PUI); ( **B** ), sonic; ( **C** ), laser; and ( **D** ), conventional needle irrigation (CNI).

In the apical third, the maximum cleanliness was achieved by group A (PUI) and group C, that is, laser activation, > group B (sonic activation), and the least cleanliness was achieved by group D (CNI) ( Fig. 2 ).

In the apical third, ultrasonic activation showcased its superior efficiency with significant *p* -value of 0.001 as compared with sonic. When comparing group A (ultrasonic activation) with group C (laser activation), it showed equivalent cleanliness with no significant difference between the two.

Between sonic and laser activation, laser activation was more efficient in achieving better debridement in apical thirds compared with sonic activation. Moreover, in the comparison between sonic activation and CNI, the average difference of –2 suggested that sonic activation was more efficient, leading to smaller scores compared with CNI in the apical third. CNI showed poor cleanliness than PUI, with a highly significant *p* -value of < 0.001.

## Discussion

In this study, intracanal heating of 3% NaOCl was performed with a heated plugger (Fi-P Heating and Packing Instrument, Woodpecker), at 180°C followed by activation as recommended by Iandolo et al, who proved in their study that intracanal heating of the irrigant at 180°C, does not increase the root surface temperature beyond 42.5°, 13 while exterior root surface temperatures above 47°C for more than 1 minute are considered to damage the Periodontal ligament (PDL). 14 The duration of heating in the present study was kept at 4 seconds recommended by Buchanan. 15

In group A, ultrasonic activation with intracanal heating of 3% NaOCl was performed through an ultrasonic tip mounted on ultrasonic activator (Ultra Smart Endo Ultrasonic Activator – COXO, China.) wherein the tip was situated at 3 mm from the working length to ensure that it does not touch the canal walls. To compensate for the deficiency that occurred as a result of volatilization and evaporation, the irrigant solution had to be renewed thrice. 16 17

In group B, sonic activation with intracanal heating was performed and polymer tip was moved in short 2 to 3 mm vertical strokes for 30 seconds after which NaOCl was replenished, and the cycle was repeated thrice. 16

## Intragroup Comparison

In the middle third, PUI and sonic activation of irrigant showed maximum cleanliness. The reason for this could be attributed to the wider canal diameter, which significantly impacts the volume and exchange of irrigant and accentuates the acoustic flow in contrast with the apical regions. 18 While in the apical third, the tip contacts the canal walls and which inhibits free oscillation reducing the displacement amplitude and thus a reduction of the debridement efficacy. 19 20 In addition to this, the apical vapor lock effect due to the entrapment of gas in the apical region may obstruct irrigant penetration.

In group C, diode laser activation was done with 980 nm diode laser (IMDSL) with a fine 200-µm fiber tip, to facilitate the effective delivery of laser light to the root canal walls. 21 Specimens treated with diode laser did not require intracanal heating as laser generates heat itself. 21 22 The 980-nm wavelength penetrates deep inside the dentinal tubules. 22 23

In the middle third, diode laser showed moderate cleanliness while in the apical third it demonstrated enhanced cleanliness. The reason for this is the closer approximation of the laser tip to the root canal walls in narrow area of the canal in the apical region, which leads to better contact, thus melting and evaporating the debris easily. 24

Group D, that is, CNI showed the lowest cleaning efficiency due to the poor exchange of fluids, which occurs only in very close proximity around the needle's tip. 25 Different studies have shown poor debridement of syringe irrigation along with poor periapical repair. 26

## Intergroup Comparison

PUI achieved significantly cleaner middle thirds compared with sonic activation. This could be attributed to the lower frequency of sonic activation (1–6 kHz), as compared with higher frequency of PUI (25–30 kHz), which causes microstreaming along with cavitation in ultrasonic that produces increased wall shear stress and enhances the breakdown of intraradicular biofilm. 27 Also, a wide variety of clinicians use ultrasonics as adjuncts in endodontic therapy. 28

On comparing sonic activation and laser activation in the middle third, laser activation was more efficient in achieving cleaner middle thirds as it has an inbuilt property of light scattering, thermal photo disruptive action in the untouched part of dentin, increased local intensity, and attenuation, which aids in light penetration deeper in dentinal tubules.

On comparing CNI with all other groups in the middle third, it was revealed that all groups outperformed CNI in achieving superior cleanliness in the middle third, as the poor cleanliness in CNI is because of vapor lock effect. 29

In the apical third, it was found that PUI showcased its superior efficiency than sonic because of the acoustic streaming component of ultrasonics and its higher frequency (25–30 kHz) resulting in multinodal formation along the length of instrument. 30

Comparing group A (PUI) with group C (laser activation), it was found that both groups showed comparable results in the apical third, with no significant difference indicating equivalent cleaning efficiency. This could be because of the inherent property of light scattering of diode lasers along with heating of irrigant, which would have caused cleaning comparable to PUI.

When comparing group A (PUI) with group D (CNI), PUI emerged as more effective in debris removal because ultrasonic activation leads to irrigant replacement at the apical third and moves the solutions apically and laterally, thus effectively cleaning debris. 4

Laser activation outperformed sonic activation in achieving cleaner apical thirds. The reason for this could be the small diameter of laser fiber along with its flexibility, which can easily enter the prepared apical root canal smoothly and demonstrate photothermal effects. 31

On comparing group C (laser activation) and group D (CNI) in apical thirds, laser activation ruled out to be more efficient compared with CNI. This is because of the flushing action on NaOCl caused by the laser beam along with the warming effect of laser radiation on the irrigating solution, which enhances the effectiveness of NaOCl. 32

Therefore, within the limitations of this study, it can be concluded that activation of 3% NaOCl enhances root canal cleanliness. Intracanal heating of 3% NaOCl with PUI outperformed all other agitation methods in removing debris in both middle and apical thirds. Diode laser activation of 3% NaOCl achieved comparable cleanliness to PUI in the apical third with no significant difference between them. Intracanal heating with sonic activation achieved reduced cleanliness in comparison to PUI and laser in the middle and apical thirds.
